# Rapid Detection of Deployment Errors for Segmented Space Telescopes Based on Long-Range, High-Precision Edge Sensors

**DOI:** 10.3390/s25113391

**Published:** 2025-05-28

**Authors:** Jisong Jiang, Xinlong Fan, Chenxu Li, Yuanyuan Tang, Shengqian Wang, Hao Xian, Mo Chen

**Affiliations:** 1 National Laboratory on Adaptive Optics, Institute of Optics and Electronics, Chinese Academy of Sciences, Chengdu 610209, China; jiangjisong@ioe.ac.cn (J.J.); fanxl@ioe.ac.cn (X.F.); yytang@ioe.ac.cn (Y.T.); sqwang@ioe.ac.cn (S.W.); xianhao@ioe.ac.cn (H.X.); 2 Manufacturing Center of Fine Mechanics, Institute of Optics and Electronics, Chinese Academy of Sciences, Chengdu 610209, China; lichenxu@ioe.ac.cn

**Keywords:** edge sensors, capacitance measurement, long-range, high-precision edge sensors, space telescope

## Abstract

The structural deformations induced by rocket launch vibrations, on-orbit thermal gradients, and gravitation fluctuations can lead to significant deployment errors for large-aperture, segmented space telescopes. As the size and number of segments increase in future telescopes, the optical-based methods for detecting deployment errors suffer from the range limitations of the millimeter scale and time-consuming processes of the month scale. To address this, we propose a new method for rapid-deployment error detection based on long-range, high-precision capacitive edge sensors. These sensors feature a measurement range of ±13 mm, with a precision better than 7.3 nm, enabling efficient and simultaneous error detection across all segments. This approach significantly reduces the time and steps required compared to traditional optical methods. Through experimental validation, the designed system demonstrated the ability to detect and correct large deployment errors and maintain co-phasing precision, meeting the stringent requirements for future space telescopes. The proposed sensor system enhances deployment efficiency, offering a viable solution for the next generation of segmented space telescopes.

## 1. Introduction

The James Webb Space Telescope (Webb) represents a new generation of large, deployable, segmented space telescopes, pioneering a new observational perspective [1]. The JWST was folded and stowed before launch. Once in orbit, the observatory deployed from its stowed state, and the optical elements realigned. Its unique design of an on-orbit, deployable, segmented primary mirror overcomes the launch size constraints imposed by rocket fairings, enabling space telescopes to achieve diameters in the 6-m class [2]. Among the four next-generation space telescopes proposed by NASA, the concepts of the Large Ultraviolet Optical Infrared Surveyor (LUVOIR) [3] and the Origins Space Telescope (OST) [4] continue to use the segmented primary mirror method.

From ground-based assembly and alignment to on-orbit deployment, factors such as the repeated positioning accuracy of the deployable structures, along with vibrations, temperature, and microgravity, will inevitably result in deployment errors in the segmented primary mirrors. Optical methods for detecting deployment errors rely on capturing the starlight from each segment using cameras. In the initial alignment errors of the JWST’s deployment, the boresight error was 5′ [5], equivalent to ~1 mm of piston error at the edge of the primary mirror. As a backup method, in the process from deployment to image stacking, mosaic imaging from Near Infrared Camera (NIRCam) A and B is used to produce a maximum mosaic coverage of ±15′ (equivalent to approximately ±3 mm of piston error at the edge of the primary mirror), which takes around 25 h to complete. When the tilt error is large, it becomes increasingly difficult to locate a bright star that is sufficiently isolated from neighboring stars. Furthermore, the number of images and the time required both increase with the square of the boresight error [6].

Currently, JWST consists of only 18 segments, and it took one month to complete the steps from primary mirror deployment to image stacking [7,8]. The timeline for the other correction steps is shown in Table 1. In future space telescopes with an increased number of primary mirror segments, using the existing methods for deployment error detection will take more time. For instance, LUVOIR-A, with a 15-m primary mirror composed of 120 hexagonal segments, will result in each star being duplicated 120 times in the mosaic image. Therefore, as the number of primary mirror segments increases, completing steps such as segment identification and image stacking using optical detection methods will become extremely challenging and time-consuming.

It is evident that as the size of segmented primary mirrors increases, optical detection methods, in terms of both measurement range and efficiency, face significant challenges in detecting deployment errors. There is an urgent need to explore new approaches for the rapid detection of deployment errors in large-scale segmented mirrors. In segmented telescopes, the detection of primary mirror co-phasing errors is not only achieved through optical means but also with the use of electronic edge sensors. These sensors are primarily employed to monitor the co-phased state of the primary mirror after optical calibration, compensating for factors such as elevation changes and temperature fluctuations, allowing the telescope to maintain a stable co-phasing state for longer observation times.

**Figure 1:**
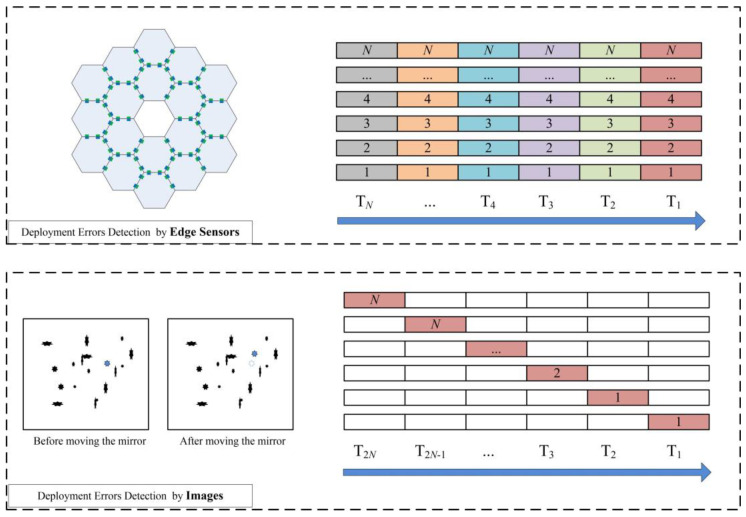
Comparison of deployment error between edge-sensor-based and optical-based detection methods.

As shown in Figure 1, the deployment error measurement method based on edge sensors enables simultaneous parallel detection and outputs the errors of all segment mirrors. Compared to optical detection methods that require the sequential traversal of each mirror, this approach significantly reduces operational steps. Assuming both methods have the same measurement rate, when the number of mirrors is *N*, the edge-sensor-based error measurement method can detect all mirrors simultaneously. In contrast, the optical-based error detection method requires the sequential movement of each mirror to determine its position, necessitating 2*N* imaging steps to complete the entire primary mirror traversal. Therefore, the edge-sensor-based detection method is more efficient.

For segmented space telescopes, the ability to detect and correct the alignment errors of the primary mirror segments after on-orbit deployment determines the final imaging quality. The JWST uses linear actuators to achieve six degrees of freedom movement for its 18 primary mirror segment assemblies (PMSAs). These actuators feature a novel stepper motor-based cryogenic two-stage design capable of 7.7 nm motion accuracy with a 21 mm range [9].

For coarse phasing, the dispersed fringe sensing method is employed, which has a measurement range limited to ±250 μm. Therefore, the relative piston error between adjacent mirror segments must not exceed this range. After coarse phasing is completed, the residual tilt error is approximately 10 pixels RMS at the stacking point (equivalent to ~7 μm of piston error at the edge of the primary mirror), and the residual piston error is approximately 0.25 μm.

It is obvious that although the JWST linear actuators have a range of motion of 21 mm, the actual deployment error of the primary mirror must be controlled within ±3 mm in tilt and within ±250 μm in the piston. This indicates that the optical system’s detection methods, based on the designed observational tasks, are limited by the inability to simultaneously ensure both field of view and precision, thus imposing stringent requirements on the deployment errors of the primary mirror [10].

In order to rapidly complete the mirror deployment to the coarse phasing steps, the measurement range of the edge sensors must surpass the motion range of the linear actuators while still maintaining a nanometer-level precision. This would allow the sensors to continue being used during subsequent co-phasing maintenance steps. Therefore, the edge sensors need to meet a measurement range of ≥21 mm and a precision of ≤7.7 nm.

Edge sensors have been successfully applied or are soon to be applied in large ground-based segmented telescopes such as KECK [11] and TMT [12] as well as to telescopes that are in the design and development stage [13]. Capacitive edge sensors, due to their simple structure, high-temperature resistance, radiation resistance [14], high resolution, and good dynamic response characteristics [15,16], have been applied in the aerospace field. For the next generation of space telescopes, research on co-phasing based on edge sensors is also being conducted [17].

Our team is dedicated to the research and development of large-aperture, segmented space telescopes. In response to the requirement for capacitive edge sensors to operate reliably during on-orbit missions exceeding ten years, we have developed a radiation-resistant capacitive edge sensor that achieves a Total Ionizing Dose (TID) tolerance of up to 1.6 × 10^9^ rad(Si) [14]. Preliminary testing also indicates that, with PT100-based temperature sensing and linear calibration, the sensor achieves thermal stability better than 1.3 nm/°C. These results demonstrate the sensor’s promising potential for space applications.

However, the current edge sensors are limited to micron-range motion and nanometer precision, making them unsuitable for detecting deployment errors during on-orbit deployment. The edge sensors and their corresponding performance parameters employed in typical ground-based segmented telescopes are presented in Table 2. It is evident that, whether capacitive or inductive, the measurement range must be limited to ensure accuracy, leading to an inherent trade-off between measurement range and precision. Based on the specified on-orbit measurement requirements, no existing edge sensors currently meet these criteria.

In response to the aforementioned challenges, we propose a method for the rapid detection of deployment errors based on long-range, high-precision edge sensors. First, the design of the edge sensors achieving a measurement range of ±13 mm is described. Next, the sensor acquisition circuit is analyzed to derive a method for dynamically adjusting the measurement range. The sensor and acquisition circuit were developed, and corresponding tests were conducted on a high-precision displacement stage. Following the theoretical analysis and experiments, it was demonstrated that the designed edge sensors with dynamically adjustable measurement range have the capability for long-range and high-precision detection of on-orbit deployment errors, effectively reducing on-orbit deployment time and the risk of exceeding error thresholds.

## 2. Method

### 2.1. Design of the Edge Sensors

The basic principle of the capacitive edge sensors is based on a parallel-plate capacitor. The capacitance can be calculated using the following equation.

(1) $$C=\frac{\varepsilon S}{d}$$

**Figure 2:**
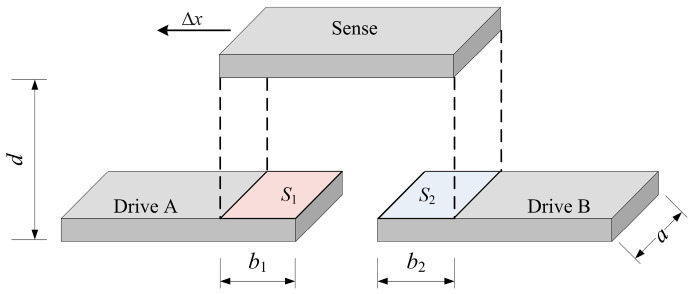
The geometry of the differential, variable-area-type capacitive sensor.

The differential, variable-area-type capacitive sensor is an optimized version of the parallel-plate capacitor structure and offers higher sensitivity. The geometry of the edge sensors is shown in Figure 2. The sensor mainly consists of drive plate A, drive plate B, and a sense plate. When the sense plate moves by a distance Δ*x*, the capacitance values *C*_*A*_ and *C*_*B*_ between the sense plate and the drive plates are as follows:

(2) $$\left\{\begin{array}{l} C_{A}=\frac{\varepsilon a\left(b_{1}+\Delta x\right)}{d}\\ C_{B}=\frac{\varepsilon a\left(b_{2}-\Delta x\right)}{d} \end{array}\right.$$

where *b*_1_ and *b*_2_ are the initial projection lengths of the sense plate on the drive plates, which are typically the same; *a* is the projection width; and *d* is the distance between the sense plate and the drive plates.

Equation (3) shows the differential capacitance value of the sensor. With the plate distance *d* and projection width *a* determined, the differential capacitance value depends only on the relative displacement Δ*x*.

(3) $$C_{diff}=C_{A}-C_{B}=\frac{2\varepsilon a}{d}\Delta x$$

**Figure 3:**
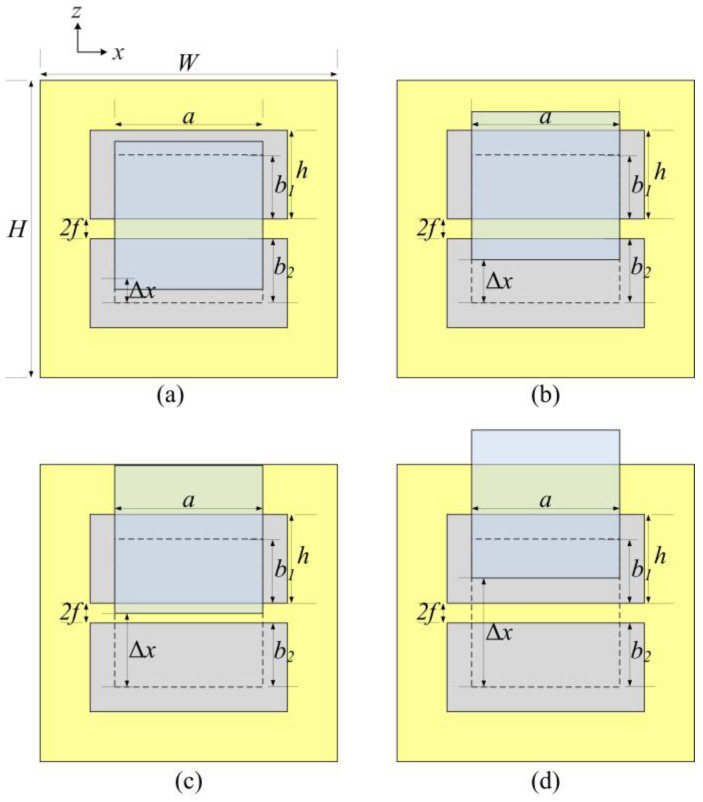
The relative position of sense plate. (**a**) Full Differential. (**b**) Partial Differential. (**c**) Fixed. (**d**) Decrease.

The actual design structure of the edge sensors is shown in Figure 3. As the sense plate moves relative to the drive plates, the projection area changes accordingly. Based on the displacement range, the formulas for calculating the differential capacitance of the sense plate at different displacement intervals are as follows.

(1) Full Differential (Curve A): When 0 < Δ*x* ≤ *h* − *b*_1_, the differential capacitance follows the corresponding relationship:

(4) $$C_{diff}=\frac{2\varepsilon a}{d}\Delta x$$

(2) Partial Differential (Curve B): *h*−*b*_1_ < Δ*x* ≤ *b*_2_.

(5) $$C_{diff}=\frac{\varepsilon a\left(h-b_{2}+\Delta x\right)}{d}$$

(3) Fixed (Curve C): *b*_2_ < Δ*x* ≤ *b*_2_ + 2*f*.

(6) $$C_{diff}=\frac{\varepsilon ah}{d}$$

(4) Decrease (Curve D): *b*_2_ + 2*f* < Δ*x* ≤ *b*_2_ + 2*f* + *h*.

(7) $$C_{diff}=\frac{\varepsilon a\left[h-\left(\Delta x-2f-b_{2}\right)\right]}{d}$$

**Figure 4:**
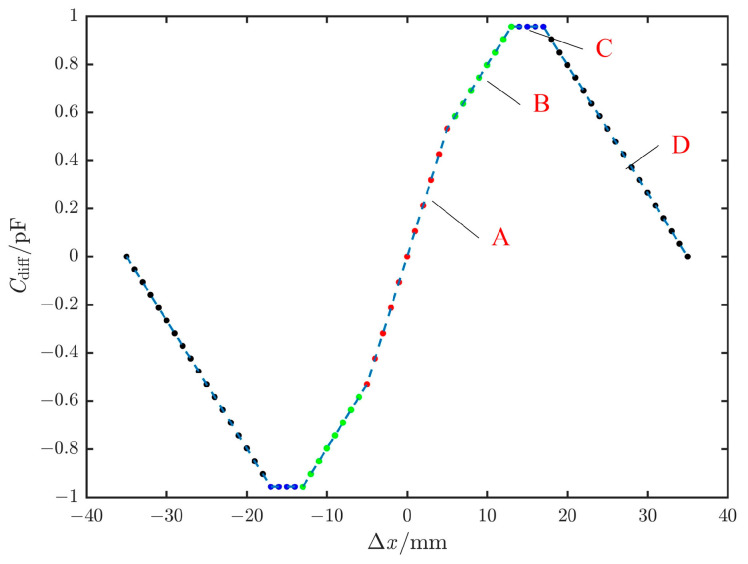
The curve of differential capacitance at different displacement intervals.

Based on previous experience [14], the design parameters of the above plates are shown in Table 3. By applying the design parameters of the plates from Table 3, the differential capacitance curve can be obtained as shown in Figure 4. When the sense plate moves in the negative direction, the differential capacitance value is the negative of the value given by the above formula.

Based on the curves, the displacement range of the sense plate is [−35 mm, 35 mm]. Within this range, the slope exhibits different linear variations between curve A and curve B, remains constant in the section of curve C, and decreases in the section of curve D. Therefore, when the relative displacement is within [−13 mm, +13 mm], the closed-loop detection and control are convergent, providing the sensor with an effective measurement range of ±13 mm.

### 2.2. Implementation of Dynamic Measurement Range

**Figure 5:**
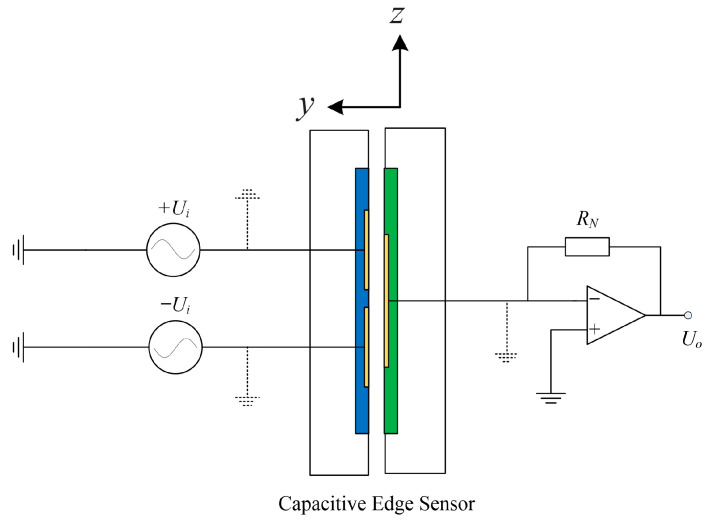
The design of the acquisition circuit. Blue: drive plate; Green: sense plate.

To detect differential capacitance changes, a capacitance signal acquisition circuit was designed, as shown in Figure 5. The circuit is composed of two Alternating Current (AC) voltage sources (+*U_i_*, −*U_i_*), the measured capacitive edge sensor (*C*_diff_), an amplifier, and a reference resistor (*R_N_*).

The differential AC voltage source *U_i_* is generated by Precision D/A Converters AD5545 (16 bits) and can generate a maximum voltage of ±4.096 V, with an integral nonlinearity (INL) within ±1 least significant bit (LSB).

The formula for a simple sine voltage is as follows:

(8) $$u=U\sin\left(wt\right)$$

And the current passing through the capacitor is as follows:

(9) $$i=C\frac{du}{dt}=CUw\cos\left(wt\right)$$

where *C* is the capacitance, and *w* = 2*πf* is the angular frequency.

According to Figure 5, the formula for the output voltage PV value *U_o_* is as follows.

(10) $$\left\{\begin{array}{l} I_{m}=C_{diff}U_{i}w\\ U_{o}=R_{N}I_{m} \end{array}\right.$$

where *R_N_* is the amplification factor of the circuit, which is derived from the equivalent of a transimpedance amplifier circuit and an AD drive circuit. And *U_i_* is the input excitation voltage PV value. By substituting (3) into (10), the relationship between the measured displacement value and the output voltage is obtained.

(11) $$U_{o}=\frac{4\pi f_{c}R_{N}\varepsilon a}{d}U_{i}\Delta x$$

where *f_c_* is the frequency of the driving sinusoidal voltage. When the plate parameters and circuit design are determined, parameters such as the driving sinusoidal voltage frequency *f*_c_ and the amplification factor of the circuit *R_N_* are generally not adjustable online and can be considered fixed values. Therefore, for different displacement intervals, the relationship between the excitation voltage *U_i_*, the measured displacement, and the output voltage can be expressed as follows.

(12) $$\left\{\begin{array}{l} U_{o}^{A}=2KU_{i}\Delta x\\ U_{o}^{B}=KU_{i}\left(h-b_{2}\right)+KU_{i}\Delta x\\ U_{o}^{C}=KU_{i}h\\ U_{o}^{D}=KU_{i}\left(h-2f-b_{2}\right)-KU_{i}\Delta x \end{array}\right.$$

(13) $$K=\frac{wR_{N}\varepsilon a}{d}$$

At the same time, the maximum output voltage *U*_max_ and minimum resolution voltage *U*_min_ of the output voltage *U_o_* are determined by the selected analog-to-digital converter (ADC). Therefore, for curves A and B within the effective measurement range shown in Figure 3, the following formulas can be derived.

(14) $$\left\{\begin{array}{l} \frac{U_{\min}}{2KU_{i}}\leq \Delta x\leq \frac{U_{\max}}{2KU_{i}}\;:\;\mathrm{Curve}\;A\\ \frac{U_{\min}}{KU_{i}}-h+b_{2}\leq \Delta x\leq \frac{U_{\max}}{KU_{i}}-h+b_{2}\;:\;\mathrm{Curve}\;B \end{array}\right.$$

It is evident that by changing the excitation voltage *U_i_*, the measurement displacement range and resolution can be dynamically adjusted. When the excitation voltage increases, the resolution improves, but the measurement range decreases.

Based on the above theoretical analysis, the equivalent design parameters for the acquisition circuit are shown in Table 4.

**Figure 6:**
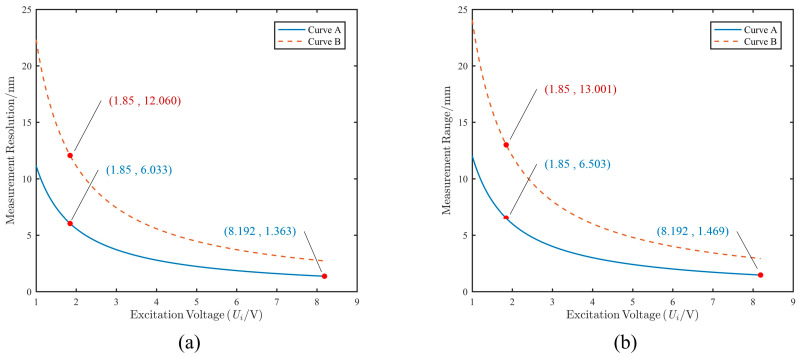
The curve of measurement displacement range and resolution for Curve A and Curve B. (**a**) Measurement resolution. (**b**) Measurement range.

By substituting the acquisition circuit parameters, the relationship between the capability of edge sensors and the excitation voltage is illustrated in Figure 6.

(1) In the range of curve A, the primary goal is to ensure detection resolution. Therefore, the excitation voltage is set to the maximum value of 8.192 V. At this setting, the detection resolution is 1.363 nm, and the measurement range is 1.469 mm.
(2) In the range of curve B, the main objective is to extend the measurement range to match the effective measurement range of the sensor. Therefore, the voltage is set to 1.85 V. At this setting, the detection accuracy is 12.060 nm, and the measurement range is 13.001 mm.
(3) Since the measurement range cannot cover a displacement of 5 mm when the excitation voltage is set to 8.192 V, the voltage is switched to 1.85 V when the displacement range exceeds 1 mm. The corresponding detection resolution decreases to 6.033 nm, and the measurement range expands to 6.503 mm.

Based on the above analysis, the excitation voltage settings are shown in Table 5. Within the displacement range of [−13 mm, 13 mm], different excitation voltages are switched according to the displacement.

## 3. Performance Evaluation

### 3.1. Experimental Setup

Based on the previous analysis, the edge sensors were designed to meet the requirements of a measurement range exceeding 21 mm and a precision better than 7.7 nm. The edge sensor plates and acquisition circuit were analyzed and designed accordingly. The simulation results shown in Figure 4 demonstrate that within a displacement range of ±13 mm, the capacitance of the edge sensors undergoes segmented changes, with the maximum capacitance value being less than ±1 pF. Additionally, an acquisition circuit was designed, which utilizes segmented excitation voltages to achieve the maximum output voltage signal envelope within the ±13 mm displacement range.

To validate the correctness of the theoretical analysis, a set of edge sensors was developed, including both the plates and the acquisition circuit. The experimental setup includes a capacitance signal acquisition circuit, the edge sensors, and two displacement stages. It is challenging to find a displacement stage that simultaneously meets the requirements of millimeter-scale displacement range and nanometer-scale resolution. Therefore, two different displacement stages were used to measure the performance of the edge sensors.

**Figure 7:**
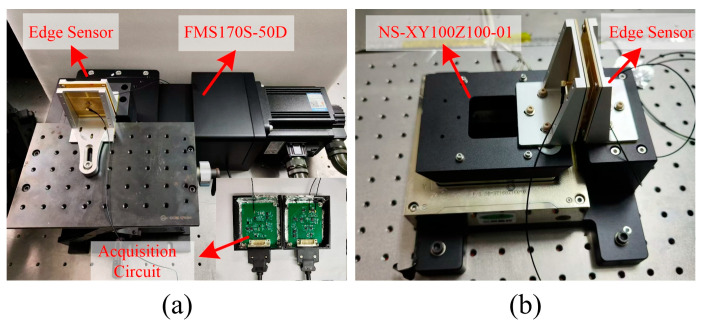
The experimental setup. (**a**) Using FMS170S-50D for large displacement measurements. (**b**) Using NS-XY100Z100-01 for high-precision measurements.

For large displacement measurements, the experimental setup is shown in Figure 7a. The displacement stage used is the FMS170S-50D, which has a minimum step size of 0.5 μm, a bidirectional repeatability accuracy of ±1.5 μm, and a total displacement of 50 mm.

For high-precision measurements, the experimental setup is shown in Figure 7b. The nanometer positioning stage NS-XY100Z100-01 is used. This stage has a closed-loop resolution of 0.5 nanometers and a travel range of 100 μm.

The laboratory temperature for performance testing was maintained at 25 °C with a humidity of 60%. Performance tests began 8 h after the sensor was powered on to ensure that the sensor reached thermal and humidity stability.

### 3.2. Differential Capacitance Response

Figure 4 shows the results from the analytical solution, which calculates the capacitance change of the sensor within ±35 mm. The displacement measurement range was divided into four segments, A to D, based on the capacitance variation trend. To validate the accuracy of the theoretical analysis, an initial trend verification was conducted using the FMS170S-50D displacement stage. However, due to the limitations of the displacement stage, the displacement range was set to ±25 mm during the experiment, with a step size of 50 μm.

**Figure 8:**
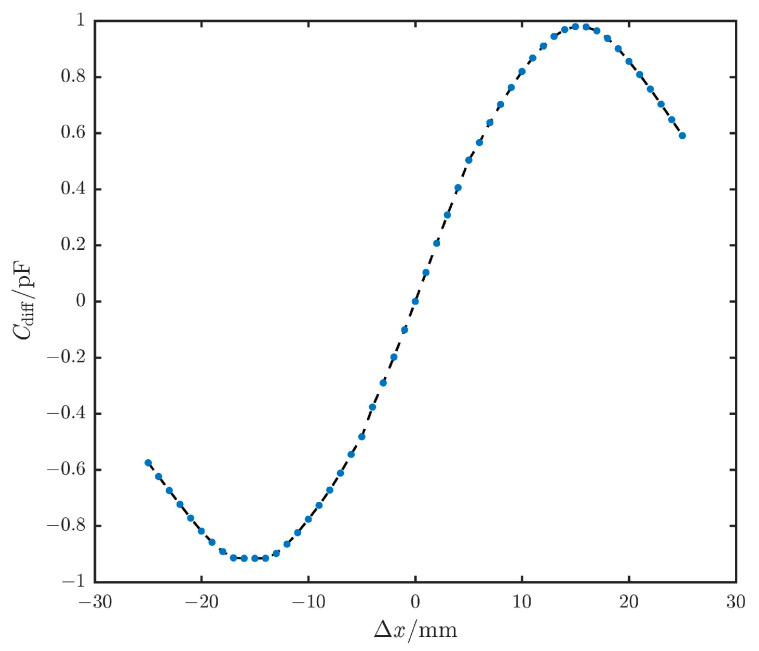
The output capacitance value curve for the ±25 mm displacement range.

The output capacitance curve obtained from the acquisition circuit is shown in Figure 8. It can be seen that the output capacitance signal maintains a good correlation with the capacitance changes in Figure 4, with a transition and decrease near ±15 mm. The experimental results demonstrated that the developed sensor achieved outcomes closely matching the theoretical analysis.

### 3.3. Displacement Detection Performance

In on-orbit operations, deployment errors can cause optical imaging spots to become distorted and potentially exceed the measurement range. The role of edge sensors in deployment error detection is twofold. First, for large deployment errors, a stepwise closed-loop iterative correction method is employed to control the linear actuators at the bottom of the primary mirror, reducing the deployment error from the coarse measurement range of 1 mm to 13 mm into the fine measurement range of ±1 mm. Second, within the ±1 mm fine measurement range, the edge sensors offer higher measurement precision, enabling the accurate detection and maintenance of co-phasing errors between adjacent segments. Therefore, the two displacement stages were used to verify whether the sensor meets the actual application requirements in terms of measurement range and accuracy within the ±13 mm displacement range.

During the displacement process, the capacitance signal acquisition circuit adjusts the voltage in real-time based on the calculated displacement, following the displacement ranges in Table 5. Specifically, when the displacement is within ±1 mm, the excitation voltage is set to 8.192 V; for ±1 mm to ±13 mm, the excitation voltage is set to 1.85 V.

For the detection performance within a ±50 μm displacement range, the NS-XY100Z100-01 is used to generate a series of displacement points from 5 nm to ±50 μm. To verify the detection performance within a ±13mm displacement range, the FMS170S-50D displacement stage is used to generate a series of displacement points from ±50 μm to ±13 mm. Through the controller, a series of displacement steps were generated on the displacement stage for 30 s, and the edge sensors operated at 1 Hz, collecting 30 points for averaging to obtain the measured displacement output.

**Figure 9:**
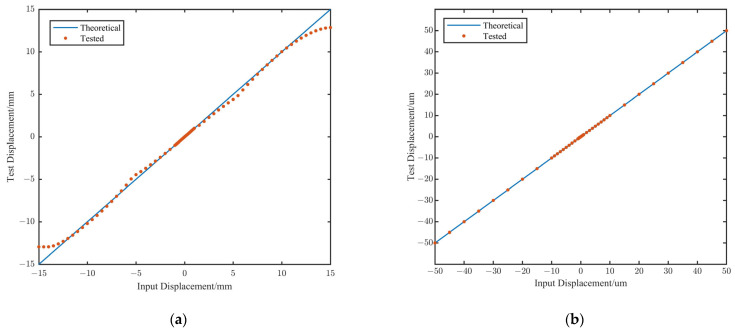
Results of displacement detection. (**a**) ±50 μm to ±13 mm. (**b**) ±5 nm to ±50 μm.

In Figure 9a, the test results for large displacements are presented. It can be observed that when the displacement exceeds 1 mm, the response curve deviates from perfect linearity. This can be attributed to several factors, including the relatively low excitation voltage, which leads to higher sensor noise, as well as edge effects and alignment errors that become more prominent at larger displacements. Further research will be conducted to address these issues. Nevertheless, the sensor maintains adequate linearity for practical applications. In cases where significant deployment errors occur, closed-loop control based on the sensors and the linear actuator can be employed to gradually reduce the deployment error, bringing it into the high-precision measurement range of ±1 mm. Figure 9b shows the test results for a displacement range of ±50 μm. The use of a piezoelectric ceramic actuator significantly improves the sensor’s linearity, making it highly suitable for the high-precision co-phasing maintenance task.

**Figure 10:**
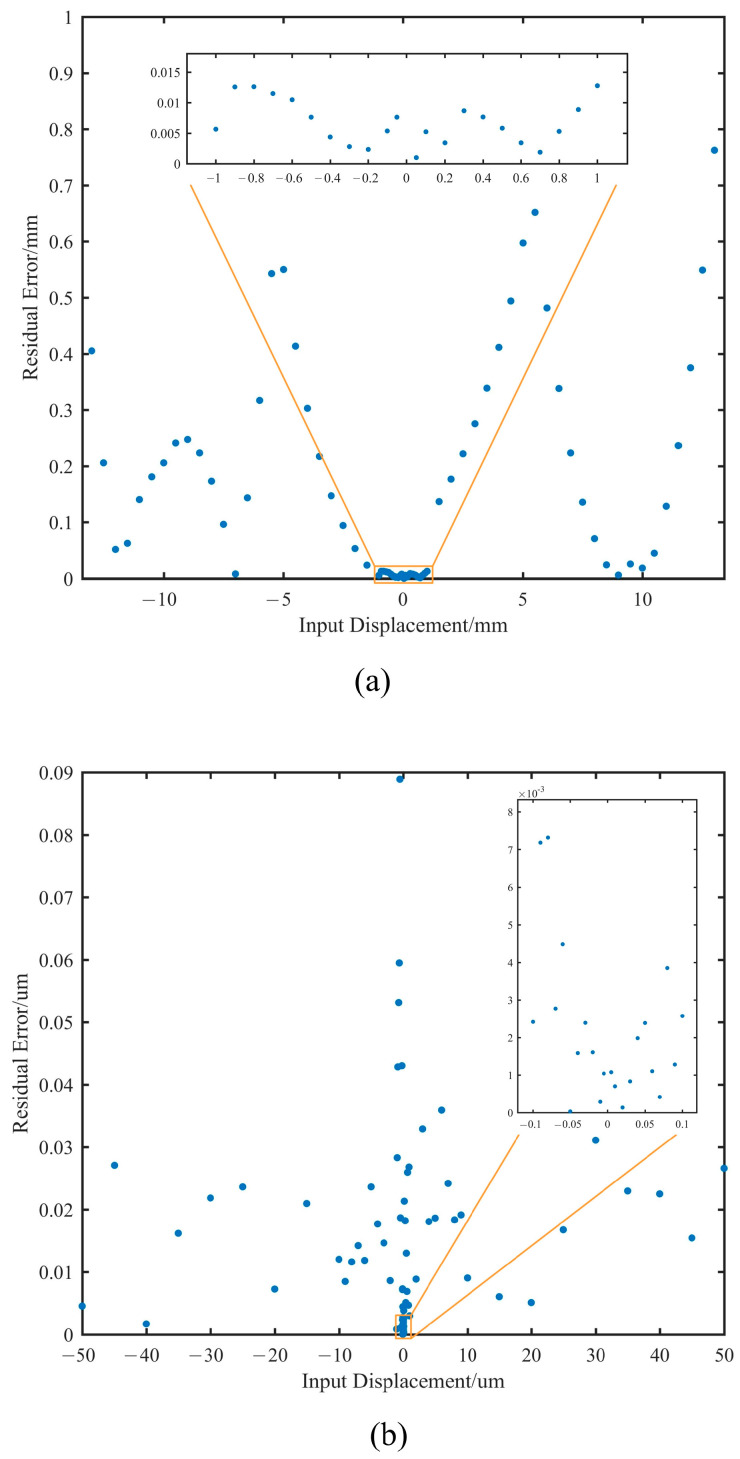
Residual errors of displacement detection. (**a**) ±50 μm to ±13 mm. (**b**) ±5 nm to ±50 μm.

The residual errors compared with the theoretical displacement values are shown in Figure 10. In different displacement ranges, the residual errors are as follows.

(1) ±1 mm to ±13 mm: The maximum residual is 0.76 mm, with an average residual of 0.24 mm.
(2) ±50 μm to ±1 mm: The maximum residual is 12.8 μm, with an average residual of 6.7 μm.
(3) ±100 nm to ±50 μm: The maximum residual is 88.9 nm, with an average residual of 14.7 nm.
(4) ±5 nm to ±100 nm: The maximum residual is 7.3 nm, with an average residual of 2.2 nm.

It can be observed that, in regions beyond the ±1 mm range, although there are locally larger errors, the deployment error can be reduced to within ±1 mm through a closed-loop iterative correction method, with measurement errors simultaneously decreasing to the 10 μm level. This implies that deployment errors can be reduced from the millimeter scale to the micrometer scale through closed-loop iteration. Within the ±50 μm range, the primary function of the edge sensors is co-phasing maintenance, and the experimental results also indicate that the sensors meet the requirement.

In the previous requirements analysis, it was determined that the edge sensors need to have a measurement range of ≥21 mm and a precision of ≤7.7 nm. Based on the actual test results, it was demonstrated that the edge sensors have a precision of 7.3 nm while also achieving a measurement range of 26 mm. This confirms the capability of the edge sensors to detect large deployment errors and co-phasing maintenance.

## 4. Conclusions

This article proposes long-range and high-precision edge sensors for detecting deployment errors in segmented space telescopes. Through the derivation, design, and experimental validation of the sensor and acquisition circuit, it is demonstrated that the sensor is capable of long range and high precision. In the long-range mode, the sensor achieves a measurement range of 26 mm with a maximum residual error of 0.76 mm. In the high-precision mode, it provides a measurement range of ±1 mm with a maximum error of less than 12.8 μm. Furthermore, in the ultra-high precision measurement range of ±100 nm, the maximum error does not exceed 7.3 nm.

Evidently, the edge sensors align their measurement range and precision with the effective displacement of the linear actuators used behind segmented primary mirrors. By employing long-range and high-precision edge sensors, the error requirements during the ground assembly and calibration of segmented space telescopes can be relaxed, reducing the difficulty of manufacturing and assembly. During on-orbit deployment, the sensors effectively match the displacement of linear actuators, facilitating a more efficient and rapid coarse phasing process. For future segmented space telescopes, the long-range and high-precision edge sensors are indispensable as effective detection instruments for deployment and co-phasing maintenance.

## Figures and Tables

**Table 1:** Timeline for JWST deployment and alignment. (licensed by Michael W. McElwain et al. under CC-BY 3.0) [7].

Activity	Prelaunch Plan	Actual
Mirror Deployments	18 January 2022	20 January 2022
Segment Identification	7 February 2022	8 February 2022
First Closed Loop Guiding	12 February 2022	13 February 2022
Segment Alignment (iteration 1)	12 February 2022	19 February 2022
Image Stacking (iteration 1)	14 February 2022	22 February 2022
Coarse Phasing (iteration 1)	21 February 2022	28 February 2022
Coarse Multi Field	22 February 2022	3 March 2022
Fine Phasing (iteration 1)	1 March 2022	8 March 2022
Fine Phasing (iteration 3)	5 March 2022	11 March 2022
Multi Field Multi Instrument Sensing 1	10 March 2022	20 March 2022
LOS Jitter Measurement	17 March 2022	21 March 2022
Multi Field Multi Instrument Sensing 2	6 April 2022	19 April 2022
OTE Alignment Complete	24 April 2022	23 April 2022

**Table 2:** The performance of the edge sensors used in typical ground-based, large-aperture, segmented telescopes.

Name	Type of Edge Sensors	Measurement Range/μm	Measurement Accuracy/nm
KECK [11]	Capacitive	±12	3
ELT [18]	Inductive	±200 ±1000	1 10
TMT [19]	Capacitive	±30	2.8
Expected	--	21,000	7.7

**Table 3:** Design parameters of the plates.

Parameters	Values	Units
Initial projection length (*b*_1_/*b*_2_)	13	mm
Projection width (*a*)	30	mm
Spacing between drive plates (2*f*)	4	mm
Single drive plate height (*h*)	18	mm
Distance of plates (*d*)	5	mm
Width of edge sensor (*W*)	60	mm
Height of edge sensor (*H*)	60	mm

**Table 4:** Equivalent design parameters of acquisition circuit.

Parameters	Values	Units
Driving voltage frequency (*f*_c_)	50	kHz
Amplification factor (R_N_)	2.04 × 10^7^	Ω
Excitation voltage (*U_i_*)	≤8.192	V
Resolution of ADC	20	bit
Maximum output voltage (*U*_max_)	8.192	V
Minimum resolution voltage (*U*_min_)	7.8	μV
Output frequency	1	Hz

**Table 5:** Excitation voltage and detection ability in different displacement intervals.

Piston/mm	Excitation Voltage/V	Measurement Range/mm	Measurement Resolution/nm
[−13, −5]	1.85	13	12.06
[−5, −1]	1.85	6.5	6.03
[−1, 0]	8.192	1.46	1.36
[0, 1]	8.192	1.46	1.36
[1, 5]	1.85	6.5	6.03
[5, 13]	1.85	13	12.06

## Data Availability

The data presented in this study are available on request from the corresponding author.
